# Automatic document classification of biological literature

**DOI:** 10.1186/1471-2105-7-370

**Published:** 2006-08-07

**Authors:** David Chen, Hans-Michael Müller, Paul W Sternberg

**Affiliations:** 1 Division of Biology and Howard Hughes Medical Institute, California Institute of Technology, Pasadena, California, USA

## Abstract

**Background:**

Document classification is a wide-spread problem with many applications, from organizing search engine snippets to spam filtering. We previously described Textpresso, a text-mining system for biological literature, which marks up full text according to a shallow ontology that includes terms of biological interest. This project investigates document classification in the context of biological literature, making use of the Textpresso markup of a corpus of *Caenorhabditis elegans *literature.

**Results:**

We present a two-step text categorization algorithm to classify a corpus of *C. elegans *papers. Our classification method first uses a support vector machine-trained classifier, followed by a novel, phrase-based clustering algorithm. This clustering step autonomously creates cluster labels that are descriptive and understandable by humans. This clustering engine performed better on a standard test-set (Reuters 21578) compared to previously published results (F-value of 0.55 vs. 0.49), while producing cluster descriptions that appear more useful. A web interface allows researchers to quickly navigate through the hierarchy and look for documents that belong to a specific concept.

**Conclusion:**

We have demonstrated a simple method to classify biological documents that embodies an improvement over current methods. While the classification results are currently optimized for *Caenorhabditis elegans *papers by human-created rules, the classification engine can be adapted to different types of documents. We have demonstrated this by presenting a web interface that allows researchers to quickly navigate through the hierarchy and look for documents that belong to a specific concept.

## Background

With so many Biology papers being published each month, researchers have a difficult time keeping track with the latest developments or finding details that were not important when the paper was published. Automated information extraction and retrieval are thus important tools for biologists (for reviews, see [1-4]). The Textpresso text-mining engine has made progress in this direction with an ontology that marks up the biological concepts within the full-texts of *Caenorhabditis elegans *papers [5]. Using a simple ontology, we found that search efficiency was improved 3-fold when looking for two uniquely named genes and a term that means an interaction. Here we explore the prospect of further utilizing the ontology to aid performance when using an algorithm to classify papers.

Within the field of information retrieval, text classification provides the means to drastically improve the efficiency of researchers. Hierarchical paper taxonomies allow users to focus on the topics and quickly locate papers of interest. In addition, taxonomies allow users to find papers that are similar. A well known taxonomy, Yahoo's Directory, analogously allows users to quickly find internet sites of interest, by first descending down a topic tree.

Many recent developments in the information retrieval field have focused on clustering ephemeral search results – live clustering of search results from general web searches [6]. Here we investigate the specific problem of clustering Biology papers. A sizable number of Biology papers are published every month (e.g., approximately 60,000 were added to PubMed in January 2006), so automated procedures are necessary for a successful categorization of papers. On the other hand, Dickman noted that machine learning algorithms did better when more human-crafted rules were involved [7]. Thus, one of the main focuses of this clustering engine is to allow more human guidance than current state-of-the-art systems to obtain higher quality results.

Support Vector Machines (SVM), with their strong theoretical foundations on structural risk minimization, have become popular tools in classification. The SVM algorithm works by learning a separating hyperplane that divides two groups of vectors. This separation requires that the text documents be represented as vectors, but this problem has been tackled in numerous classification and clustering algorithms. Generally, each word in the vocabulary of the corpus becomes a dimension, and a vector represents the number of occurrences of the respective words in the document. Word-stemming is utilized so that words such as "cell" and "cells" are counted together. Joachims showed that Support Vector Machines could classify papers more accurately than previous algorithms [8]. Support Vector Machines work well for text classification since there are many words in the vocabulary, yielding a high-dimensional vector space. At the same time, each paper might only use a small subset of the thousands of words in the vocabulary of the corpus. Support Vector Machines are thus well suited for such document vectors that are sparse but contain dense concepts (i.e., the words that are present in a document are important).

While Support Vector Machines are powerful and allow the user much control of the classification, creating many subcategories and finding associated training papers would be prohibitively expensive in terms of human effort. Thus, another method must also be employed to more autonomously create categories and assign the documents to them. Conventional clustering algorithms, such as k-means, separate documents by vector representations, similar to those used in SVM, and then attempt to label the clusters. In contrast to such algorithms, recent research on clustering has focused on creating meaningful labels for the clusters, often by extracting phrases to represent underlying concepts [9]. Such algorithms are an active field of research, but many of the most successful algorithms are closed source. For example, Vivisimo has one of the best commercial clustering engines, capable of extracting a hierarchy of concepts and classifying a variety of documents, from search engine results to abstracts of scientific reports [10]. While many of the recent systems focus on clustering on-the-fly search results, our system performs the classification as a background task. This allows our system to utilize system resources more extensively without runtime as a constraint. For example, we can utilize the full text of the papers instead of just the abstracts. The human-based guidance that our setup involves also provides more control of the produced taxonomy, to help ensure that the results match what a user would expect.

Beil et al. described an algorithm to hierarchically classify documents based on frequent term sets [11]. Their system provided an intuitive way for users to understand the contents of the clusters. For example, the top clusters for a corpus of documents on the beach may be: "sun", "fun", "beach", and "surf." The second layer of clusters would be: "sun, fun", "surf, beach", etc. Such annotations marked a step forward from conventional clustering algorithms that would first cluster the papers and then attempt to label the clusters, usually producing labels that are difficult for humans to understand.

## Results

**Figure 1:**
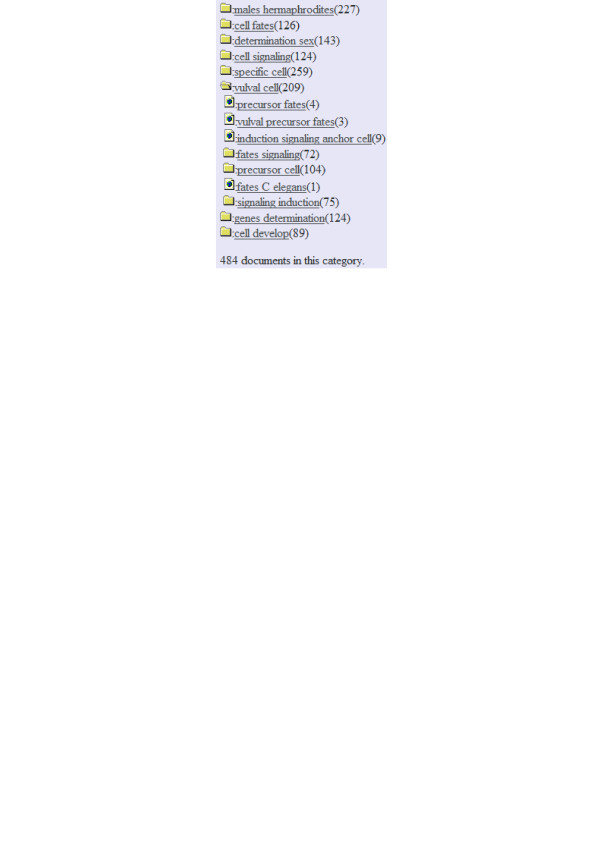
**An example of the clustering results from the Sex Determination category**. An intuitive interface allows users to quickly locate the topic of interest. The topics listed were generated automatically during the phrase-based clustering step.

We developed a document classification engine that can classify papers into a topic-based hierarchy. The engine is currently optimized for *Caenorhabditis elegans *papers by using human-created rules. The rules are in the form of a list of terms associated with each topic. The engine classifies all articles with the full-text available from Textpresso, which currently has over 7000 such papers. By combining two different methods, the classification results are more useful than conventional algorithms while minimizing the amount of manual curation needed. Support Vector Machine-based classifiers assign the documents into nine primary categories, and a phrase-based clustering process creates a hierarchy of up to 200 subcategories for each primary category and labels the papers with these finer descriptions. The classification engine outputs the taxonomy as a large number of HTML files, which allow users to intuitively parse through the hierarchy to find the papers belonging to a biological concept. Figure 1 presents an example of the interface that users browse through to find topics of interest.

### Classification by support vector machine

The initial classification is done by Support Vector Machine, which assigns each paper into at least one of the nine main categories. The nine categories were taken from the chapters of WormBook: Genetics/Genomics, Molecular Biology, Cellular Biology, Sex Determination, Developmental Control, Signal Transduction, Neurobiology and Behavior, Ecology and Evolution, and WormMethods [12]. The Germline chapter from WormBook was excluded, since the category would contain too few papers (please see the Methods and Materials section for detailed analysis). These nine categories divide the nematode literature in a manner with which biologists are familiar, guaranteeing that the top layer of classification matches the expectations of users.

Support Vector Machines learn a separating hyperplane that separates two groups of vectors. Following conventional practice when representing text documents as vectors, each dimension of the vector space represents a word in the corpus. When creating the vector representations, words that are not useful are skipped. Most of the rules, such as skipping stopwords and words that occur in too many or too few of the documents, apply to all text domains. A few domain-specific rules are utilized: when counting the number of occurrences of words in a documents, words that are in human-created lists of important words are multiplied by a boosting factor to increase the classification performance. The domain-specific rules for skipping words and the boosting improvement are described in Methods.

Since a paper can belong to more than one of the nine categories, the multi-class classification is done with nine runs of one-against-all classification for each paper. One-against-all refers to the fact that each SVM classification decides whether a document belongs to a category or does not belong. In addition, the SVM step forces each paper to belong in at least one category, since the assumption is that all papers in the *C. elegans *Textpresso corpus discuss the biology of nematodes.

The training set currently consists of 226 examples, but a training document may be a positive example for more than one category. Using 10-fold cross-validation, we achieved micro-averaged precision of 69.57% and micro-averaged recall of 65.17% (see the evaluation section for definitions of recall and precision). 10-fold cross-validation refers to dividing the training set into ten groups of 22 documents and then treating a different group as the test set during each of the ten runs. While the cross-validation performance looks low, the decision to allow a paper to belong in multiple categories would give even a human difficulty in classification, since there is no strict threshold for the extent to which a paper must discuss a category before being assigned to it.

The distribution of the papers, which is shown in Table 1, appears reasonable to the expected number of articles that should discuss each topic. For example, many papers discuss cellular processes or structure, so the Cell Biology category is expected to be large. The WormMethods category is somewhat larger than expected because it also functions as a catch-all for worm papers that did not belong elsewhere. After SVM, the mean number of categories per paper is 1.195 with standard deviation of 0.438.

The output of the SVM step serves directly as data to feed into the next step. Since there can be more than a thousand papers assigned to a category after SVM and such a number could easily be overwhelming, users are not given an interface to browse only the results of the SVM process. Instead, the clusters produced by phrases found within each category serve to further divide the papers within each of the nine main topics.

### Clustering by frequent phrases

The key step in clustering is to find phrases that can serve as descriptive labels for the clusters. From informal analysis, phrases of two and three words were found to be descriptive and represented many Biology topics. Thus, for each of the nine categories, the most frequent two and three word phrases are automatically mined and considered as the possible clusters for the respective categories.

A distinguishing aspect of clustering Biology literature is which types of phrases are preferred. In general searches, proper nouns, such as names of people and places, are highly descriptive and useful to quickly locate specific information. For Biology researchers, in contrast, there already exist tools to locate papers by specific authors and biological topics are preferred over names. Thus, the mark-up provided by Textpresso is used to automatically decide which phrases are likely to represent concepts. Furthermore, each of the nine categories has a list of manually curated words that should be emphasized in the clusters. For example, the Genetics category emphasizes the words "transposon", "homolog", "repeats", etc. These lists of words are used as human guidance to help the algorithm choose which phrases to use as potential clusters. The lists vary from 35 words for the smaller categories up to 83 words for the WormMethods category, so the combined human effort to construct all the lists is minimal. Both the lists of words and the rules that utilize the Textpresso markup are not necessary to the function of the system. They should be modified for enhanced clustering quality if using this system on a new corpus that has been marked up by Textpresso.

After the program automatically checks that the phrases are not mentioned in too many papers in the category, parent-child relationships are found via a subsumption algorithm similar to what Sanderson and Croft describe (also described in the Materials and Methods section) [13]. Each cluster can have at most one parent to ensure an acylic graph.

At the risk of creating extraneous clusters, the preference is that there exist enough clusters so that the leaves of the hierarchy are sufficiently descriptive to be useful. All papers are assigned to at most four clusters in each category, and papers that do not contain any of the phrases for a category are excluded from the classification for that category.

Despite the customizations that tailor the engine to cluster Biology literature, the overall process can create a topic hierarchy from general search results. As a proof of principle, a program to cluster the top 150 results for a given query from Yahoo was created. The rules specific to *Caenorhabditis elegans *papers are ignored (i.e., the ontology is not used), and a few rules specific to snippets from search engines are inserted: words such as "free" and "welcome" are added to the list of words to skip. An online interface for the clustering engine is available [14].

### Testing

We used the Reuters 21578 test-set as a benchmark to compare the quality of the clustering engine against previously published results. This set consists of 21,578 news articles from 1987 that were later indexed by humans and is freely available for download [15].

Beil et al. used a subset of 8654 articles from the Reuters 23157 set [11]. This subset consists of those articles that were manually tagged to belong to exactly one topic.

A common way to measure clustering quality among a hierarchy is to use the F-measure. First, the recall and precision for a given topic *T*_*k *_and cluster *C*_*j *_must be defined. The recall refers to the ability to retrieve all expected results while precision represents the portion of returned results that are correct. For each cluster and topic, the documents tagged with that topic represent the set of expected results and the cluster acts as the results returned.

Let *n*_*j*,*k*_ denote the number of documents in cluster *C*_*j *_that also belong to topic *T*_*k*_; that is, the number of correct results.

Recall(Tk,Cj)=nj,k#Tk     (1)
 MathType@MTEF@5@5@+=feaafiart1ev1aaatCvAUfKttLearuWrP9MDH5MBPbIqV92AaeXatLxBI9gBaebbnrfifHhDYfgasaacH8akY=wiFfYdH8Gipec8Eeeu0xXdbba9frFj0=OqFfea0dXdd9vqai=hGuQ8kuc9pgc9s8qqaq=dirpe0xb9q8qiLsFr0=vr0=vr0dc8meaabaqaciaacaGaaeqabaqabeGadaaakeaaieGacqWFsbGucqWFLbqzcqWGJbWycqWGHbqycqWGSbaBcqWGSbaBcqGGOaakcqWGubavdaWgaaWcbaGaem4AaSgabeaakiabcYcaSiabdoeadnaaBaaaleaacqWGQbGAaeqaaOGaeiykaKIaeyypa0ZaaSaaaeaacqWGUbGBdaWgaaWcbaGaemOAaOMaeiilaWIaem4AaSgabeaaaOqaaiabcocaJiabdsfaunaaBaaaleaacqWGRbWAaeqaaaaakiaaxMaacaWLjaWaaeWaceaaieqacqGFXaqmaiaawIcacaGLPaaaaaa@4A25@

Precision(Tk,Cj)=nj,k#Cj     (2)
 MathType@MTEF@5@5@+=feaafiart1ev1aaatCvAUfKttLearuWrP9MDH5MBPbIqV92AaeXatLxBI9gBaebbnrfifHhDYfgasaacH8akY=wiFfYdH8Gipec8Eeeu0xXdbba9frFj0=OqFfea0dXdd9vqai=hGuQ8kuc9pgc9s8qqaq=dirpe0xb9q8qiLsFr0=vr0=vr0dc8meaabaqaciaacaGaaeqabaqabeGadaaakeaaieGacqWFqbaucqWFYbGCcqWGLbqzcqWGJbWycqWGPbqAcqWGZbWCcqWGPbqAcqWGVbWBcqWGUbGBcqGGOaakcqWGubavdaWgaaWcbaGaem4AaSgabeaakiabcYcaSiabdoeadnaaBaaaleaacqWGQbGAaeqaaOGaeiykaKIaeyypa0ZaaSaaaeaacqWGUbGBdaWgaaWcbaGaemOAaOMaeiilaWIaem4AaSgabeaaaOqaaiabcocaJiabdoeadnaaBaaaleaacqWGQbGAaeqaaaaakiaaxMaacaWLjaWaaeWaceaaieqacqGFYaGmaiaawIcacaGLPaaaaaa@4E50@

where #*T*_*k *_denotes the total number of documents with this topic and #*C*_*j *_denotes the number of documents that belong in this cluster.

The F-measure for the given pair of topic *T*_*k *_and cluster *C*_*j*_, is then

F−measure(Tk,Cj)=2*Recall*PrecisionRecall+Precision     (3)
 MathType@MTEF@5@5@+=feaafiart1ev1aaatCvAUfKttLearuWrP9MDH5MBPbIqV92AaeXatLxBI9gBaebbnrfifHhDYfgasaacH8akY=wiFfYdH8Gipec8Eeeu0xXdbba9frFj0=OqFfea0dXdd9vqai=hGuQ8kuc9pgc9s8qqaq=dirpe0xb9q8qiLsFr0=vr0=vr0dc8meaabaqaciaacaGaaeqabaqabeGadaaakeaacqWGgbGrcqGHsislcqWGTbqBcqWGLbqzcqWGHbqycqWGZbWCcqWG1bqDcqWGYbGCcqWGLbqzcqGGOaakcqWGubavdaWgaaWcbaGaem4AaSgabeaakiabcYcaSiabdoeadnaaBaaaleaacqWGQbGAaeqaaOGaeiykaKIaeyypa0ZaaSaaaeaacqaIYaGmcqGGQaGkcqqGsbGucqqGLbqzcqqGJbWycqqGHbqycqqGSbaBcqqGSbaBcqGGQaGkcqqGqbaucqqGYbGCcqqGLbqzcqqGJbWycqqGPbqAcqqGZbWCcqqGPbqAcqqGVbWBcqqGUbGBaeaacqqGsbGucqqGLbqzcqqGJbWycqqGHbqycqqGSbaBcqqGSbaBcqGHRaWkcqqGqbaucqqGYbGCcqqGLbqzcqqGJbWycqqGPbqAcqqGZbWCcqqGPbqAcqqGVbWBcqqGUbGBaaGaaCzcaiaaxMaadaqadiqaaGqabiab=ndaZaGaayjkaiaawMcaaaaa@707F@

The range of the F-measure falls between 0 to 1, with 1 indicating the best quality for a cluster. The overall F-measure for the entire hierarchy is the weighted average among the topics, choosing the cluster for each topic that gives the highest F-measure, i.e., it is the summation over all the topics, adding the F-measure from the best cluster for that topic multiplied by the number of documents that are known to belong in that topic divided by the total number of documents.

Beil et al. obtained an overall F-value of 0.49 [11]. The clustering engine from this project obtained an improved F-value of 0.55, but the key advantage conferred by our system is that the produced clusters, labeled with phrases, are easier to understand than clusters annotated by term sets. For example, one of the top-level clusters our system produces on the Reuters set is "last year", with child clusters such as "trade surplus" and "trade deficit". These types of relationships between phrases would not be possible under Beil's system because their system finds term sets, and the next layers must include the previous layers: as illustrated in the Background section, the top clusters for a corpus of documents on the beach may be: "sun", "fun", "beach", and "surf." The second layer of clusters are then constrained to be: "sun, fun", "surf, beach", etc.

As another comparison, Larson et al. obtained F-values that varied around 0.6 using a clustering engine that is designed to run quickly and scale linearly with the number of documents [16]. While they obtained fairly good results from the numerical measurement, their cluster annotations consist of the features that were considered important during clustering and may not necessarily be understandable to a human. These tests do not utilize the Textpresso ontology with the Reuters text.

As a test of the SVM performance, classification on the top 10 Reuters categories was performed. This set consists of the 6490 training articles, and 2545 testing samples. The SVM classification obtained a micro-averaged F1 score of 0.946 and macro-averaged F1 score of 0.874. These scores slightly exceed the results that Debole and Sebastiani obtained on this set with SVM classification [17]. As Debole and Sebastiani describe, this Reuters subset is the easiest because it contains the most training examples. Such a test, however, is a good measure for the top-level classification that SVM provides in our clustering engine, since each category should have a substantial number of training examples. The competitive SVM classification performance indicates that this initial classification stage performs as well as state-of-the-art methods.

## Discussion

### Accomplishments

This clustering system provides an effective way to classify documents into a taxonomy, with category descriptions that are easily understandable by humans. The benchmarking experiments indicate that the clustering results are competitive with state-of-the-art algorithms. Such a taxonomy with useful cluster labels allows novices in the field to quickly discover the key concepts among the papers while enabling experts to browse through the hierarchy and quickly locate papers discussing a specific topic. The classifications also allow researchers to locate similar papers, by finding papers that contain the same concept. Both, the provision of key concepts of a research field as well as the ability to find similar papers, are not easily achieved by simply entering a set of keywords into a search engine. The software, along with source code, is available for download [18].

The human-provided guidance helps ensure higher quality clustering results. For example, the Sex Determination category provides the following for the top layer of choices: "hermaphrodite male", "sex determination", "development gene", "development cells", "cells fate", "vulval cells", "anchor cells", and "cells male." As a comparison, the search for "sex determination Caenorhabditis elegans" on ClusterMed, Vivisimo's engine to cluster up to 500 titles and abstracts from pubmed, yielded the top clusters as "Fem", "Dosage compensation", "Behavior", "Gld-1", "Mab-3", "Fog, Germ cells", "Caenoharbdities Elegans Sex-Determining Gene Tra-2", "cDNA Sequence", "Translational control", and "Fish Medaka". Compared to Vivisimo's results, our labels emphasize concepts compared to gene names, which should be more useful for researchers trying to explore the field.

The immediate goal of the project was to provide a way to classify *Caenorhabditis elegans *literature, but the process to classify text in another domain is fairly straightforward. The curator must first choose the primary categories, create lists of important words in each of these categories, and then identify training papers for these categories. Domain-specific rules may be needed in the code to specify which words should be skipped in the vector representations. For increased SVM performance, the SVM parameters should also be tuned for these categories, but this step can be automated by the software system once the training papers are found. If an ontology has been used to markup the text in the new domain, the rules used in the clustering step should be modified although an ontology is not required.

The unique combination of Support Vector Machines with phrase-based clustering allows the creation of a topic taxonomy that is more guided, and thus, of higher quality than current state-of-the-art methods. At the same time, the amount of human guidance required is kept limited to finding the initial training set for the SVM step. Other steps of human intervention such as finding lists of boosted words or rules for using the Textpresso markup are minimal compared to this step.

Besides the utility of the entire system, the individual components of the project might be useful. As demonstrated with the clustering engine on Yahoo search results, documents or snippets from a general source may be used to construct a topic hierarchy. The phrase-based clustering algorithm, while currently optimized for a specific group of papers, can be adapted to a variety of different types of documents. An ontology is required for neither SVM classification nor phrase-based clustering. On-the-fly clustering of search results can also be performed on *Caenorhabditis elegans *literature, but the clustering engine is currently not optimized for such usage.

### Areas for improvement

The SVM process provides acceptable performance, but further slight modifications may allow better performance. For example, recent reports have indicated superior text classification performance when using transductive SVM, which creates the separating hyperplane while maximizing the margin to both the training and testing vectors, compared to the inductive SVM that the system currently uses [19]. Another concern is the creation of the training set, finding the optimal precision and recall that can be obtained, and which documents to train on to minimize the work needed in making the training set. Schohn and Cohn developed an approach called active learning, an iterative process of identifying papers to add to the training set [20].

The clustering engine, with few published algorithms to compare to, is highly experimental. More human guidance could be implemented when choosing possible phrases or creating the hierarchy. Concepts may be found to be synonyms by looking in a knowledge source, such as the Metathesaurus included in the UMLS [21]. The UMLS also includes data sources that organize topics into a hierarchy. For example, the Medical Subject Headings included in UMLS can relate apoptosis as a child concept of cell death, which is a child concept of cell physiology, and so forth. Thus, by integrating these kinds of relations that have already been created by expert curators into the probabilistic method currently used, a better hierarchical tree could be produced.

## Conclusion

We have presented a simple but effective two-step method to categorize a corpus of biological papers. It consists of a support vector machine component as well as a novel phrase-based clustering algorithm. The method automatically generates clusters with labels that are intuitive and understandable to humans. It is amenable to human intervention and modification such as hand-crafted rules, but can also be used in an unsupervised environment. This method performs competitively when compared to similar algorithms.

## Methods

All software was written in Java to allow reusability of the written code. While Java may have longer run-times than comparable languages, this choice was considered appropriate since the classification is done as a background task on the server. Thus, as long as the classification does not take excessively long (such as more than three days, which is a specification easily met for a corpus of seven thousand papers), the runtime speed was not a concern. A cronjob runs the classification engine weekly, and another cronjob copies the classification results to the public HTML folder. Thus, there is never any downtime from the user's perspective, but there may be times when the classification results are slightly out of date, when the newest papers have not been categorized yet. The classification for the *C. elegans *corpus can be accessed online through the Textpresso website [22].

**Figure 2:**
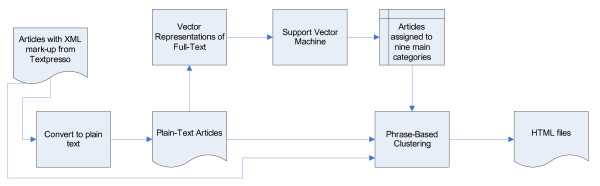
**Overview of the classification process**. Full-text papers are taken from the Textpresso corpus and processed via SVM and phrase-base clustering. The end result is a large set of html files displaying the paper taxonomy.

Figure 2 illustrates the classification process. The xml files of the papers are converted into plain-text, and these plain-text files are then used to represent the documents as vectors, which are then fed into the SVM machinery, resulting in nine primary categories. The assignments of the papers into the categories are then used during phrase-based clustering, which uses both the plain-text files and the XML files.

### Support vector machine

The plain-text papers are used as inputs to the SVM classification. In order to optimally form vector representations from the text documents, the words must first be parsed, and then non-useful words are skipped. Words are found by splitting at each space or hyphen character. Using domain-specific and general rules, certain words may be skipped: a list of 191 stop-words, which is applicable for most text classification tasks, contains prepositions and other such words that are not useful. Words that consist of just digits are also skipped. In addition, words that are less than three characters long can usually be skipped, but the words that often have special biological significance such as "X", "Y", "XY","XO", "XX" and "G" are kept even though they are short.

The words are mapped to feature stems by lower-casing the characters and applying the Porter Stemming algorithm [23]. The counts of the feature stems are recorded for each document, and the frequency counts of the features are used in the vector representations. Features that occur in more than 95% of the documents or are used less than three times are considered uninformative and are not used in forming the document vectors. Each of the remaining feature stems represents a dimension in the vector space. The feature counts for the document vectors are then weighted by the TF•IDF scheme.

feature−weight(tk,dj)=tCount(tk,dj)*(1+log⁡#docsdCount(tk))     (4)
 MathType@MTEF@5@5@+=feaafiart1ev1aaatCvAUfKttLearuWrP9MDH5MBPbIqV92AaeXatLxBI9gBaebbnrfifHhDYfgasaacH8akY=wiFfYdH8Gipec8Eeeu0xXdbba9frFj0=OqFfea0dXdd9vqai=hGuQ8kuc9pgc9s8qqaq=dirpe0xb9q8qiLsFr0=vr0=vr0dc8meaabaqaciaacaGaaeqabaqabeGadaaakeaacqWGMbGzcqWGLbqzcqWGHbqycqWG0baDcqWG1bqDcqWGYbGCcqWGLbqzcqGHsislcqWG3bWDcqWGLbqzcqWGPbqAcqWGNbWzcqWGObaAcqWG0baDcqGGOaakcqWG0baDdaWgaaWcbaGaem4AaSgabeaakiabcYcaSiabdsgaKnaaBaaaleaacqWGQbGAaeqaaOGaeiykaKIaeyypa0JaemiDaqNaem4qamKaem4Ba8MaemyDauNaemOBa4MaemiDaqNaeiikaGIaemiDaq3aaSbaaSqaaiabdUgaRbqabaGccqGGSaalcqWGKbazdaWgaaWcbaGaemOAaOgabeaakiabcMcaPiabcQcaQiabcIcaOiabigdaXiabgUcaRiGbcYgaSjabc+gaVjabcEgaNnaalaaabaGaei4iamIaemizaqMaem4Ba8Maem4yamMaem4CamhabaGaemizaqMaem4qamKaem4Ba8MaemyDauNaemOBa4MaemiDaqNaeiikaGIaemiDaq3aaSbaaSqaaiabdUgaRbqabaGccqGGPaqkaaGaeiykaKIaaCzcaiaaxMaadaqadiqaaGqabiab=rda0aGaayjkaiaawMcaaaaa@7904@

where *tCount *represents how often feature *t*_*k *_occurs in document *d*_*j *_and *dCount *represents how many documents contain *t*_*k*_. This is a conventional weighting scheme for SVM that emphasizes those terms that occur less frequently in the corpus. A noticeable performance gain can be obtained by boosting the feature-weights of those words that are important in each category by a factor of 5. This performance increase comes for free since the lists of boosted words should be prepared for the phrase-based clustering.

The document vectors are then normalized to unit length 1, so that abnormally long or short documents do not adversely affect the training process. The nine SVM models for each category are then created with LIBSVM [24], a library that provides an implementation of SVM in Java. The model for each category is trained using cost and gamma parameters that have been tuned for that category, and shrinking is turned on. These parameters are found using a tool included with LIBSVM that performs a logarithmic grid search, with cost ranging from 2^-5 ^to 2^15^, multiplying by four for each step, and gamma ranging from 2^-15 ^to 2^3^, also multiplying by four for each step. Shrinking reduces the number of operations for the training process with minimal loss in accuracy. The training set is represented by a tab-delimited file that indicates to which categories each training paper is known to belong. The papers in the training set were found with keyword-based searches in Textpresso. For example, a sample of the top results for a search of "genetics" were added in the training set as examples of genetics papers, although some of these papers may have also been examples for other categories.

After the training is complete, all documents in the corpus are classified one at a time. While classifying a paper, the probability that a paper belongs to each category is also calculated. If a paper is classified as not belonging to any of the nine categories, the paper is assigned to the category with the lowest probability of not belonging to it. This method allows a paper to belong to multiple categories and guarantees that each paper will be assigned to at least one category. The algorithm that implements probability estimates for SVM classification is described by Wu, et al [25]. The implementation has been slightly modified so the same seed is used when randomly assigning the documents into five groups for computing probability estimates; this ensures that results are the same from run to run without changing the purpose of the function provided by LIBSVM.

### Analysis of SVM decisions

Instead of using the bag-of-words model, an alternative approach was to use the mark-up provided by Textpresso, which can parse for n-grams. For example, the phrase "MAP kinase" could be used as one dimension in the vector model instead of the bag-of-words model, where the phrase would be split into the words "map" and "kinase." Another issue was that the full-text files provided by Textpresso include the references at the bottom of each paper. Thus, a good fraction of the words in each document were lists of author names and titles of cited papers. Finally, the boosting factor for words that were listed to be important was incremented from 0 to 9. All three issues were thoroughly investigated by performing runs with the varying parameters, and the results are presented in Table 2, with the standard deviations of the average precision and recall included in parentheses. The runs labeled untrimmed are the full-text papers on the bag-of-words model while the trimmed runs are also on the bag-of-words model, but trimming the paper on the last instance of "literature cited", "references", or "acknowledgement" and which is at least after 2/3 of the paper. The tests show that these citations actually help SVM performance, so they are kept in the production system. This makes sense since papers that share a topic tend to cite the same papers. In addition, automatically trimming the citations may not always work as intended since papers may delineate their references without words and only visual separations such as white space, which our PDF-parsing tool can not interpret.

A paired T-test of the F1 value between XML and untrimmed results has a probability of .8474, indicating that there is an 84.7% chance that the observed differences could be seen if using XML and untrimmed papers were the same. A paired T-test is appropriate since the varying boosting factor provides 10 pairs of data points. Hence, it is not statistically significant whether XML or the untrimmed articles are used. The untrimmed papers was decided to be the source since it is faster to parse than XML and the classification performance is not dependent on the quality of the markups provided by the ontology (which could be important if applying this classification system on a domain outside *Caenorhabditis elegans*). In addition, the optimum value for the boost factor on untrimmed papers is 5.

### Clustering by phrases

To assist in mining phrases that describe concepts, each category has its own list of boosted words, and these lists also act as a form of human guidance to choose descriptive and useful phrases. These boosted words are crucial in deciding how to label potential clusters, or else the clusters in all categories would be dominated by the same common phrases in nematode biology. Most papers, for instance, mention gene expression and proteins, and phrases associated with these topics are frequently found. For the lists currently used for *Caenorhabditis elegans *literature, most of the words are the sub-chapters from WormBook. The table of contents for Developmental Control contains the subchapters "Asymmetric cell division and axis formation in the embryo", "translational control of maternal RNAs", "Gastrulation in *C. elegans*", etc. Except for words that are not specific to the category such as "*C elegans*", the words in the titles of the subchapters are included in the lists of important words. The lists of boosted words are provided in [Additional file 1].

The phrase extraction process begins by using the XML markup from Textpresso to locate 200 phrases that should be descriptive. Phrases consist of 2 or 3 consecutive XML elements. If any of these elements are labeled as pronoun, modality, or intention, then this is considered a junk element list. In addition, at least one element must be considered useful, which is met if it is labeled as "function", "entity_feature", "process", "organism", "method", "gene", "transgene", "allele", or "cell". The purpose of these requirements is that the phrases contain a useful concept and to avoid phrases such as "using with". These rules would need to be modified when clustering a text corpus marked up with a different ontology.

In addition, general rules are applied: phrases that contain repeating words or too many stopwords are skipped, and the remaining candidate element-sets are counted in all the documents of the category. Sets of the same word stems are counted together so that phrases such as "neurotransmitter transporters" and "transport neurotransmitters" are counted together, but the phrase is labeled by the form it was seen the first time.

In order to choose 200 phrases that could form the clusters in the category, the phrases are scored to emphasize phrases that are descriptive for the current category. The cover of a phrase is defined, as was done by Beil et al., to be the set of documents that contain at least one instance of that phrase [11]. In addition, the nested cover of a phrase is the union of its own cover and the cover of all its child phrases. The mined phrases are weighted according to their cover size multiplied by the number of boosted words in the phrase. The presence of a word marked as 'gene" by Textpresso increments the count of boosted words by .1 since phrases that describe genes are frequently important in biology.

After automatically extracting and choosing the top 200 phrases, the remaining procedure for generating the hierarchy is general for all text domains. The trimmed, plain-text representations of all the papers in the category are loaded into memory since the XML sources are no longer needed. Phrases with a cover greater than 80% of the possible documents are excluded since they are too general to be useful.

The subsumption processes is based on the assumption that two phrases that co-occur in the same sentence often must have some type of relationship. The parent-child relationships are found via a simple probabilistic model. Let x and y be two phrases that have been extracted and are possible clusters with a hierarchical relationship. Let P(x|y) be the ratio of sentences containing y that also contain x. Now, if the limit of P(x|y) approaches 1 and the limit of P(y|x) approaches 0 with respect to all sentences in the corpus, then this implies that x can occur without y, but y is found every time x occurs. This implies that y is a child concept of x. The algorithm first checks that y does not contain more documents than x before assigning x as a parent of y. In addition, the algorithm will not assign the child to a potential parent if the parent already has 8 or more children unless this is the worst possible parent for the child. The hierarchical assignments begin with the x,y that give the greatest P(x|y) and continue until the probability becomes less than 1%. P(x|y) is computed as the count of combining both phrases divided by the count of phrase y. For performance reasons, the calculation of P(x|y) does not require scanning the entire corpus; instead, we only need to check the documents that are known to contain at least phrase y to find the count of phrase y and when counting combined phrases, only the intersection of the covers of both phrases needs to be checked.

After creating the hierarchy, all documents in the category must be assigned into the clusters, which are each labeled by a phrase. For each document, each possible cluster is weighed proportionally by the number of occurrences of the corresponding phrase and inversely proportionally to the nested cover of the cluster after the creation of the hierarchy. Each document is then assigned to the four clusters with the highest weights. The choice of four clusters is an arbitrary decision to prevent documents from belonging to too many clusters and overwhelming the user.

To encourage user functionality, certain post-processing steps are used on the tree. In particular, clusters that are considered useless are removed. These include phrases that ended up with zero documents or leafs that end in a stopword. In addition, if the first layer contains fewer than eight categories, then the branch with the largest cover is moved up from the largest top layer. This last step is done to encourage user friendliness, to ensure that there exist a reasonable number of choices available.

### Analysis of primary categories

While all the nine categories are the primary sections from WormBook, the Germline section was excluded as a category because it would contain too few papers. To confirm this, an experiment was done with two runs of SVM classification. For the first run, 18 papers (the top results from Textpresso after searching for "germline" and manually checking their relevance) were labeled as instances of the Germline category. In addition, a subset of the existing training set was added, so that all 10 categories would have approximately the same number of training examples. For the second run, all Germline examples were labeled as Sex Determination (although two papers already were marked as instances of Sex Determination in the first run). The experiment shows that if Germline were a separate category, both the Sex Determination and Germline category would be much too small compared to the other categories as displayed in Table 3. Thus, these two categories are treated the same, since the Germline is a concept frequently related to Sex Determination. All SVM training was done with cost of 5 and gamma of 1.0.

## Authors' contributions

DC devised and developed all aspects of the algorithm, performed the computational experiments and analyzed them, implemented the web-interface and its underlying data-processing and wrote the manuscript. HMM initiated the project, provided the Textpresso database and infrastructure, suggested ideas and edited the manuscript. PWS edited the manuscript, provided materials and supervised the project. All authors read and approved the final manuscript.

## Supplementary Material

Additional file 1 **List of boosted words for the nine primary categories**. Each category contains its own list of words that are given special emphasis during SVM and phrase-based clustering. Click here for file

## Figures and Tables

**Table 1:** Distribution of training examples and SVM output among the nine main categories.

	Number of examples in training set	Number assigned
Genetics	36	769
Molecular Biology	31	702
Cellular Biology	45	1532
Sex Determination	23	475
Developmental Control	42	1198
Signal Transduction	28	912
Neurobiology and Behavior	27	1214
Ecology and Evolution	21	505
WormMethods	39	1286

**Table 2:** Comparison of boosting and using XML on SVM performance with 10-fold cross validation on training set

	Average precision (sigma)	Average recall (sigma)	F1 score
XML (boost 0)	0.6328 (0.0913)	0.5836 (0.0961)	0.6072
Untrimmed (boost 0)	0.6387 (0.0953)	0.5875 (0.1028)	0.6120
Trimmed (boost 0)	0.6305 (0.1005)	0.5874 (0.1016)	0.6082
XML (boost 1)	0.6527 (0.0970)	0.6173 (0.0877)	0.6345
Untrimmed (boost 1)	0.6531 (0.0864)	0.6214 (0.0918)	0.6369
Trimmed (boost 1)	0.6351 (0.0965)	0.6173 (0.0996)	0.6261
XML (boost 2)	0.6673 (0.0790)	0.6486 (0.0775)	0.6578
Untrimmed (boost 2)	0.6566 (0.0655)	0.6316 (0.0761)	0.6438
Trimmed (boost 2)	0.6533 (0.0618)	0.6314 (0.0834)	0.6422
XML (boost 3)	0.6800 (0.0770)	0.6556 (0.0755)	0.6676
Untrimmed (boost 3)	0.6722 (0.0546)	0.6419 (0.0622)	0.6567
Trimmed (boost 3)	0.6472 (0.0478)	0.6315 (0.0593)	0.6393
XML (boost 4)	0.6780 (0.0857)	0.6414 (0.0897)	0.6592
Untrimmed (boost 4)	0.6843 (0.0624)	0.6522 (0.0745)	0.6678
Trimmed (boost 4)	0.6571 (0.0640)	0.6241 (0.0766)	0.6402
XML (boost 5)	0.6820 (0.0908)	0.6456 (0.1035)	0.6633
Untrimmed (boost 5)	0.6957 (0.0746)	0.6517 (0.0655)	0.6730
Trimmed (boost 5)	0.6708 (0.0817)	0.6207 (0.0863)	0.6448
XML (boost 6)	0.6994 (0.0781)	0.6594 (0.0758)	0.6788
Untrimmed (boost 6)	0.6926 (0.0859)	0.6485 (0.0798)	0.6698
Trimmed (boost 6)	0.6680 (0.0929)	0.6172 (0.0966)	0.6416
XML (boost 7)	0.6863 (0.0737)	0.6382 (0.0784)	0.6614
Untrimmed (boost 7)	0.6865 (0.0851)	0.6415 (0.0864)	0.6632
Trimmed (boost 7)	0.6732 (0.0869)	0.6207 (0.0987)	0.6459
XML (boost 8)	0.6703 (0.0709)	0.6176 (0.0886)	0.6429
Untrimmed (boost 8)	0.6817 (0.0682)	0.6276 (0.0704)	0.6535
Trimmed (boost 8)	0.6843 (0.0759)	0.6245 (0.0939)	0.6530
XML (boost 9)	0.6801 (0.0748)	0.6142 (0.0906)	0.6455
Untrimmed (boost 9)	0.6807 (0.0722)	0.6167 (0.0724)	0.6471
Trimmed (boost 9)	0.6749 (0.0775)	0.6070 (0.0963)	0.6392

**Table 3:** Comparison of number of assigned documents when germline is available as a category

	With Germline as a category		Without Germline as a category	
	
	Training examples	Documents assigned	Training examples	Documents assigned
Genetics	25	736	25	722
Molecular Biology	20	611	20	683
Cellular Biology	21	1101	21	1277
Sex Determination	18	205	34	398
Developmental Control	22	796	22	721
Signal Transduction	20	922	20	757
Neurobiology and Behavior	20	1431	20	1401
Ecology and Evolution	18	495	18	470
WormMethods	23	1070	23	1154
Germline	18	118		

## References

[B1] Andrade MA, Bork P (2000). Automated extraction or information in molecular biology. FEBS Lett.

[B2] De Bruijn B, Martin J (2002). Getting to the (c)ore of knowledge: Mining biomedical literature. Int J Med Inf.

[B3] Staab S, (editor) (2002). Mining information for function genomics. IEEE Intell Syst.

[B4] Jensen LJ, Saric J, Bork P (2006). Literature mining for the biologist: from information retrieval to biological discovery. Nature Reviews Genetics.

[B5] Muller HM, Kenny EE, Sternberg PW (2004). Textpresso: An ontology-based information retrieval and extraction system for biological literature. PLoS Biol.

[B6] Ferragina P, Gulli A (2004). The anatomy of a hierarchical clustering engine for Web-page, news and book snippets. Proceedings of the Fourth IEEE International Conference on Data Mining.

[B7] Dickman S (2003). Tough Mining. PLoS Biol.

[B8] Joachims T (1998). Text Categorization with Suport Vector Machines: Learning with Many Relevant Features. Proceedings of the 10th European Conference on Machine Learning.

[B9] Hammouda K, Kamel M (2004). Efficient Phrase-based Document Indexing for Web Document Clustering. IEEE Transactions on Knowledge and Data Engineering.

[B10] Vivisimo: Clustering – automatic categorization and meta-search software. http://www.vivisimo.com/.

[B11] Beil F, Ester M, Xu X (2002). Frequent term-based text clustering. Proceedings of the Eighth ACM SIGKDD.

[B12] WormBook: Online Review of C. elegans Biology. http://www.wormbook.org/.

[B13] Sanderson M, Croft B (1999). Deriving concept hierarchies from text. Proceedings of the 22nd ACM SIGIR.

[B14] Online interface to clustering engine of Yahoo search snippets. http://www.textpresso.org/webcluster.

[B15] Reuters-21578 text categorization test collection distribution 1.0. http://www.daviddlewis.com/resources/testcollections/reuters21578/.

[B16] Larsen B, Aone C (1999). Fast and effective text mining using linear-time document clustering. Proceedings of the Fifth ACM SIGKDD.

[B17] Debole F, Sebastiani F (2005). An analysis of the relative hardness of Reuters-21578 subsets. Journal of the American Society for Information Science and Technology.

[B18] Download site for automatic classification software. http://www.textpresso.org/clustering-software.

[B19] Joachims T (1999). Transductive Inference for Text Classification using Support Vector Machines. Proceedings of the 16th ICML.

[B20] Schohn G, Cohn D (2000). Less is more: Active Learning with support vector machines. Proceedings of the 17th ICML.

[B21] Unified Medical Language System. http://www.nlm.nih.gov/research/umls/.

[B22] Textpresso: An information retrieval and extraction system for biological literature. http://www.textpresso.org/.

[B23] Porter MF (1980). An algorithm for suffix stripping. Program.

[B24] LIBSVM: a library for support vector machines. http://www.csie.ntu.edu.tw/~cjlin/libsvm.

[B25] Wu T, Lin C, Weng RC (2004). Probability estimates for multi-class classification by pairwise coupling. J Machine Learning Research.

