# Supplementary material for: Automatic document classification of biological literature
Source: BMC Bioinformatics. 2006 Aug 7;7:370. doi: 10.1186/1471-2105-7-370 (PMC1559726; doi:10.1186/1471-2105-7-370)
Supplement: Additional file 1 — List of boosted words for the nine primary categories. Each category contains its own list of words that are given special emphasis during SVM and phrase-based clustering. [file 1471-2105-7-370-S1.doc]

# Supplementary Materials

The boosted words for each category can be inputted as phrases, but they are parsed as individual words.

### Genetics/Genomics:

Karyotype

ploidy

gene dosage

essential

redundant redundancy

duplication

complementation

suppression

enhancer

mosaic

mutation

genomics

gene structure

classification

repeated motif

families

superfamily

transposon

transposable element

mitochondrial

repetitive DNA

repeat families

functional genomic data

homolog

noncoding

coding

map

ortholog

region

evolving

developmentally

sequence

complex expression pattern

repress

chromosome

protein

predicted

amino acid

site

### Cell Biology:

Transcription mechanism

transcriptional regulation

DNA repair

alternative splicing

trans-splicing operon

trans splicing

premessenger RNA

snRNP

snRNA

pre-mRNA

RNAi mechanisms

RNA interference

double stranded

double-stranded

microRNA

regulate translation

mRNA decay

ubiquitin

degredation

RNA binding protein

oxidation reduction

NADPH

enzyme activity

DNA damage

inducing expression

overexpression

polypeptide

catalyst

protease

proteolytic

binding domain

noncoding

coding

translate

tumor supressor

reading frame

primer

misfolded

structure

### Molecular Biology:

division

fusion

cuticle

basement membrane

intracellular trafficking

cytoskeleton

extracellular matrix

cadherin superfamily

gap junctions

microtubule

kinetochore

anaphase

prophase

metaphase

telophase

cytokinesis

dynein

kinesin

sperm motility

filament

myosin

intermediate filament

sarcomere assembly

actinin

mitosis

meiosis

chromatin

nucleus

nuclear envelope

protease

organelle

cytoplasm

proteoglycan

transglutaminase

calcium

enzyme activity

oxidation reduction

mitochondrial

heparan sulfate

oxidation reduction

NADPH

tumor supressor

disulfide isomerase

lamina

lamin

laminan

titin

vesicle

vesicular

oxidative stress

homeostasis

atpase

### S**ex Determination:**

sex determination

male female development

hermaphrodite

fate specification

anchor

vulva precursor

somatic

germline germ

evolution

x chromosome dosage compensation

x-linked

lateral signal

lin-12 notch

progeny

xx

xo

larvae l1 l2 l3 l4 stage

hox

vulval induction

self-fertile fertile

### Developmental Control Mechanisms:

developmental control

asymmetric cell division

axis formation

embryo

larva

stage

translational control maternal RNA

gastrulation

epidermal morphogenesis

organogenesis

organ formation

embryological variation

programmed cell death

apoptosis

E cell specification

notch signal

blastomere

endoderm

mesoderm

### Signal Transduction:

Map kinase signaling

mek

mapk

rtk

mitogen

calcium

calmodulin

ras

receptor tyrosine kinase

kinase

transforming growth factor

tgf

Lin-12 notch

Wnt signal

Chemoreceptor families

Heterotrimeric g-proteins

gtpase

nuclear hormone receptor

immune response

hedgehog signaling network

nuclear localization

cascade

pathway

ligands

specificity

downstream effector

atp

### Neurobiology and Behavior:

neurobiology

behavior

axon

nervous system

synapse

acetylcholine

potassium calcium channel

synaptic function

GABA

glutamate receptor

monoamine

neuropeptide

neurotransmitter

ethanol

locomotion

mating behavior

egg-laying behavior

defecation

aggregation

chemosensation

mechanosensation

neurodegeneration

neuronal disease

neuron

associative learning

serotonin

olfactory learning

neuroregeneration

### Nematode Evolution and Ecology:

Evolution

ecology

parasite

nematode diversity

nematode phylogeny

phylogenetic relationships

Rhabditid

genome evolution

evolution of development

natural variation

bacteria

ecology

microbial pathogens

population

pleiotropy

mutation rate

aging

life span

fitness cost

random

body size

ploidy

oxygen regulator tension

### WormMethods:

forward genetics

mapping

genetic markers

compound mutants

supressor mutations

synthetic enhancer mutations

epistasis

mosaic analysis

maintenance

genetic balancers

reverse genetics

RNAi methods

injection

soaking

agar plates

liquid culture

mutant libraries

knockouts

visualizing

protein interactions

culture medium

reporter gene fusion

in situ hybridization

dna microarray

transformation

microinjection

RNA interference

transgenic strain

assay conditions

blot

functional genomics

lab technique

ideal organism

model organism

toxicity test toxic

deletion mutagenesis

living dead distinguishing nematode

dye

axenic culture

growth supplement

indefinite cultivation

prolong

control

surgery

neurosurgery

cloning vector
